# Frecuencia de terceros molares retenidos con relación al biotipo facial. un estudio transversal

**DOI:** 10.21142/2523-2754-1002-2022-105

**Published:** 2022-06-27

**Authors:** Taylor Augusto Zeta Rodríguez

**Affiliations:** 1 División de Radiología Bucal y Maxilofacial, Universidad Científica del Sur. Lima, Perú. taylor_zeta@hotmail.com Universidad Científica del Sur División de Radiología Bucal y Maxilofacial Universidad Científica del Sur Lima Peru taylor_zeta@hotmail.com

**Keywords:** frecuencia, terceros molares retenidos, biotipo facial, frequency, retained third molars, facial biotype

## Abstract

**Objetivo:**

Identificar la frecuencia de terceros molares retenidos con relación al biotipo facial utilizando estudios 2D (panorámicos y cefalométricos) de pacientes que acudieron al centro de imágenes Zeta-Exmed en el periodo de noviembre de 2018 a diciembre de 2021, en la ciudad de Lima, Perú.

**Metodología:**

El tipo de estudio de esta investigación fue observacional, descriptivo, transversal y retrospectivo. Se evaluaron 6000 casos, cada uno compuesto de una imagen radiográfica panorámica y una cefalométrica, ambas tomadas al paciente en la misma fecha, de las cuales solo fueron incluidos en la investigación un total de 150 casos, por cumplir con los criterios de selección. Las mediciones sobre la frecuencia de terceros molares retenidos con relación al biotipo facial se realizaron por un investigador entrenado y calibrado, utilizando el *software* Nemoceph para los trazados cefalométricos. Se utilizó para el análisis estadístico el programa SPSS versión 1.0.0.1401 (SPSS Inc., EE. UU.) y la prueba chi cuadrado (p < 0,05).

**Resultados:**

De los 150 casos revisados, 91 correspondieron al sexo femenino (60,66%) y 59 al sexo masculino (39,33%), lo que dio como resultado que el biotipo dolicofacial se presentó con mayor frecuencia en estos, con un total de 27 casos (45,80%), mientras que, en mujeres, el biotipo braquifacial fue el de mayor frecuencia, con un total de 35 casos (38,50%). Asimismo, del total de terceros molares retenidos evaluados (403 piezas), 141 se presentaron en casos con biotipo dolicofacial, seguido del biotipo mesofacial con 139 piezas y, finalmente, el biotipo braquifacial con 123 piezas. Respecto de la evaluación de los terceros molares retenidos en relación con su posición y biotipo facial, se encontró 63 3MR en posición mesioangular y 28 en posición distoangular en dolicofacial, seguidos de 48 piezas en posición vertical en braquifacial.

**Conclusiones:**

No existe una relación significativa entre la cantidad de terceros molares retenidos y su posición, en relación con el biotipo facial, pero sí respecto a la relación entre el sexo y el biotipo facial, pues los varones presentan un biotipo dolicofacial con mayor frecuencia y las mujeres el biotipo braquifacial.

## SUMMARY

**Objective:**

This study aims to determine the frequency of retained third molars in relation to facial biotype, the data were collected by using the patients’ 2D studies (panoramic and cephalometric) attending the imaging center ZETA-EXMED in the period November 2018 to December 2021, in the city of Lima, Peru.

**Methods:**

The type of study used for this research are observational, descriptive, cross-sectional, and retrospective. For the present study, 6000 cases were evaluated, each case consists of one panoramic radiographic image and one cephalometric image, both taken to the patient on the same date, of these, only 150 cases that met the selection criteria were included in this study. Measurements about frequency of retained third molars in relation to facial biotype were done by a trained and calibrated researcher, using NemoCeph software for the cephalometric tracing. The statistical analysis was done using SPSS program version 1.0.0.1401 (SPSS Inc., USA) and the Chi square test (p <0.05).

**Results:**

Of the 150 cases observed, 91 were women (60.66%) and 59 were men (39.33%). According to the results, dolichofacial biotype had the highest frequency among males with a total of 27 cases (45.80%), while the brachyfacial biotype was more prevalent among females, with a total of 35 cases (38.50%). In addition, out of the total number of retained third molars evaluated (403 pieces), 141 were present in cases with dolichofacial biotype, followed by the mesofacial biotype with 139 pieces and finally the brachyfacial biotype with 123 pieces. Regarding the evaluation of retained third molars according to their position and facial biotype, 63 retained third molars were found in mesioangulated position and 28 retained third molars in dolichofacial / distoangular position, followed by 48 pieces in the vertical position in brachyfacial.

**Conclusions:**

There is not significant association between the quantity of retained third molars and their positions, in relation to facial biotype, although there is a significant association between facial biotype and the patient's sex, so it was concluded that Dolichofacial biotype has the highest frequency in males, while the Brachyfacial biotype is seen more frequently among females.

## INTRODUCCIÓN

La importancia de la imagenología en odontología es realizar un examen sistemático, por lo que resulta de suma importancia para realizar una correcta evaluación y así llegar a un diagnóstico adecuado del estado del paciente, ya que proporciona información que, en muchos casos, no es posible obtener únicamente con el examen clínico. Esto permite la identificación de diversas patologías y alteraciones óseas y dentarias, observadas con frecuencia durante la atención realizada por el cirujano dentista ^1^^-^^4^.

Los terceros molares, son las últimas piezas dentales en erupcionar y pueden llegar a presentar una gran cantidad de alteraciones, algunas relacionadas con su ubicación o posición. Pueden estar ligadas con el aumento de la densidad del hueso que lo rodea o por tejidos blandos adyacentes muy queratinizados, además de la angulación de piezas dentales contiguas o de la misma pieza, por lo que las fuerzas eruptivas serán insuficientes para superar estas barreras. La clasificación de Winter sirve para evaluar la inclinación del diente retenido en relación al eje del segundo molar. Se le considera diente retenido a la pieza cuya corona se encuentra aún dentro del hueso, pero el desarrollo de sus forámenes apicales ha llegado a su fin, por lo cual se afirma que ha fallado en su proceso de erupción para llegar a su posición ideal ^5^^-^^13^.

Por otro lado, el biotipo facial está basado en variantes de proporción músculo esqueletal que tienen relación con la dirección del crecimiento craneal; esto se encuentra conectado con factores hereditarios, ambientales o sistémicos que pueden dar como resultado valores predominantes entre las dimensiones verticales y horizontales del rostro. Además, el diámetro y el espacio de las arcadas dependen de la forma de la cara, por lo que está en relación a la probabilidad de encontrar terceros molares retenidos (3MR), sobre todo en pacientes dolicofaciales caracterizados por presentar el rostro alargado en sentido vertical y estrechamiento de ambas arcadas. El análisis de Ricketts y el índice de VERT son los estudios de elección y los más confiables para la determinación del biotipo facial ^14^^-^^24^.

La importancia de evaluar 3MR y contrastarlos con el biotipo facial es tener un diagnóstico temprano para efectuar un plan de tratamiento adecuado y así predecir el desarrollo de la pieza dental, así como, en los casos que fuera necesario, evaluar el abordaje quirúrgico en cirugía maxilofacial. En ortodoncia se considera un factor importante la existencia de suficiente estructura ósea; si los terceros molares son extraídos antes de la cirugía, beneficiaria ampliamente en la remodelación ósea; así mismo, en la evolución del paciente para continuar con el tratamiento planeado sea de movimiento dental o abordaje quirúrgico ^25^^-^^35^.

No hay suficiente evidencia en la literatura que soporte la prevalencia entre el biotipo facial y los 3MR, sin embargo, algunos estudios han determinado que el biotipo facial sí está relacionado con esto. Es importante que se siga evaluando el tema para ver si efectivamente esta relación de variables existe o no. Por lo tanto, el propósito del presente estudio pretende determinar la frecuencia de 3MR con relación al biotipo facial por medio de estudios imagenológicos 2D, a fin de obtener un diagnóstico temprano de 3MR para su posterior evaluación y plan de tratamiento en pacientes con un biotipo facial determinado, lo que beneficia a los cirujanos dentistas en sus tratamientos.

## MATERIALES Y MÉTODOS

El estudio fue de tipo observacional, descriptivo, transversal y retrospectivo y se desarrolló usando imágenes radiográficas digitales de pacientes atendidos en el centro especializado en radiología bucal y maxilofacial Zeta-Exmed en el periodo noviembre del 2018 a diciembre del 2021, en Lima, Perú, a los cuales se les realizó una radiografía panorámica y una radiografía cefalométrica, siendo justificadas bajo la necesidad de evaluación general, previa al tratamiento de ortodoncia. 

La muestra estuvo conformada por 6000 casos de radiografías digitales, cada uno compuesto por una radiografía panorámica y una cefalométrica, que fueron tomadas en la misma cita con el equipo radiográfico de la marca PointNix, modelo 800S HD Plus (fabricado en Corea). La muestra fue determinada siguiendo los criterios de selección previamente establecidos. El análisis de imágenes panorámicas para evaluar la posición de los terceros molares retenidos de ambas arcadas de acuerdo con la clasificación de Winter, se realizó con el *software* original del equipo, CDX, y el análisis cefalométrico de Vert para determinar el biotipo facial, se realizó con el *software* NemoStudio Fall Edition 2020 (NemoCeph).

Todas las mediciones fueron realizadas por un investigador previamente calibrado y capacitado por un especialista en radiología bucal y maxilofacial con 5 años de experiencia, además de un especialista en ortodoncia de más de 10 años de experiencia en la evaluación de los trazados cefalométricos. 

La calibración intraobservador se desarrolló utilizando el coeficiente de correlación intraclase (CCI); las variables fueron medidas en 2 momentos, con un intervalo de 5 minutos, hasta obtener valores mayores a 0,7 en todas las mediciones realizadas, y la parte estadística se realizó con ayuda del programa SPSS para Windows versión 25.0, buscando identificar asociaciones entre el biotipo facial y la presencia del tercer molar retenido mediante la prueba de chi cuadrado, así como la influencia de las covariables; sexo, presencia de terceros molares y biotipo facial, con un nivel de significancia de p < 0,05.

## RESULTADOS

De un total de 6000 casos, cada uno compuesto por una radiografía panorámica y una cefalométrica, se obtuvieron un total de 150 casos que cumplieron con los criterios de selección establecidos, de los cuales, 91 correspondieron al sexo femenino (60,66%) y 59 al sexo masculino (39,33%).

La edad mínima fue de 18 años y la máxima de 36 años en los casos incluidos. Se encontró que el biotipo dolicofacial fue el más frecuente en hombres, con 27 casos (45,80%), mientras que las mujeres representaron una mayor frecuencia al biotipo braquifacial con 35 casos (38,50%). La reseña detallada de estos resultados esta descrita en la Tabla 1.

**Tabla 1:** Distribución del biotipo facial según sexo

Sexo	Biotipo Facial					p
	Braquifacial		Mesofacial	Dolicofacial	Total		
Masculino	n	15	17	27	59	0,031*
	%	25,40%	28,80%	45,80%	100,00%	
Femenino	n	35	33	23	91	
	%	38,50%	36,30%	25,30%	100,00%	
Total	n	50	50	50	150	
	%	33,30%	33,30%	33,30%	100,00%	

Test de chi cuadrado

* significativo

Se observó que dentro de los 150 casos (100%), hubo 403 3MR, 48 de las cuales estaban en posición vertical correspondientes al biotipo braquifacial alcanzando la mayor frecuencia en esta distribución. También se encontró en las 3MR mesioanguladas que 63 correspondían al biotipo dolicofacial tomando el valor más alto en esta distribución. También se encontró que las 3MR distoanguladas dieron como resultado un total de 28 estando principalmente en el biotipo dolicofacial. Además, se encontraron 3MR en posición horizontal, invertidas y en posición ectópica con los valores de 8, 7, y 12 3MR respectivas a los biotipos dolicofacial, braquifacial y mesofacial, respectivamente.

La asociación de 3MR con el biotipo facial dio como resultado que la suma de todos los dientes correspondientes a cada biotipo facial fueron 123 3MR correspondientes al biotipo braquifacial, 139 3MR al biotipo mesofacial, y en el biotipo dolicofacial se encontraron 141 3MR.

En la Tabla 2 se observa la asociación entre el biotipo facial y el número de 3MR, lo que dio como mayor resultado los casos con biotipo facial braquifacial, mesofacial y dolicofacial los que presentaban 2 3MR (con valores de 17, 18, 18 respectivamente a los biotipos faciales anteriormente mencionados), pero los resultados no fueron significativos. La asociación entre el biotipo facial y la posición de 3MR por cuadrante esta descrita en la Tabla 3, resaltando que no hubo diferencia significativa (p > 0,05).

**Tabla 2:** Asociación entre el biotipo facial y el número de terceras molares retenidas.

Biotipo Facial		Número de treceras molares retenidas					p
		1	2	3	4	Total		
Braquifacial	n	12	17	7	14	50	0,208
	%	24,00%	34,00%	14,00%	28,00%	100,00%	
Mesofacial	n	5	18	11	16	50	
	%	10,00%	36,00%	22,00%	32,00%	100,00%	
Dolicofacial	n	3	18	12	17	50	
	%	6,00%	36,00%	24,00%	34,00%	100,00%	

Test de chi cuadrado

**Tabla 3:** Asociación entre el biotipofacial y la posición de la tercera molar retenida por cuadrante.

Biotipo Facial		Posición de la tercera molar retenida en el cuadrante I						p
		Vertical	Mesioangulada	Distoangulada	Invertida	Ectópica	Total		
Braquifacial	n	13	3	8	1	0	25	0,281
	%	52,00%	12,00%	32,00%	4,00%	0,00%	100,00%	
Mesofacial	n	11	3	13	0	3	30	
	%	36,70%	10,00%	43,30%	0,00%	10,00%	100,00%	
Dolicofacial	n	13	5	13	0	0	31	
	%	41,90%	16,10%	41,90%	0,00%	0,00%	100,00%	
Biotipo Facial		Posición de la tercera molar retenida en el cuadrante II					p	
		Vertical	Mesioangulada	Distoangulada	Ectópica	Total		
Braquifacial	n	13	3	11	1	28	0,899	
	%	46,40%	10,70%	39,30%	3,60%	100,00%		
Mesofacial	n	12	3	12	3	30		
	%	40,00%	10,00%	40,00%	10,00%	100,00%		
Dolicofacial	n	15	5	14	1	35		
	%	42,90%	14,30%	40,00%	2,90%	100,00%		
Biotipo Facial		Posición de la tercera molar retenida en el cuadrante III						p
		Vertical	Mesioangulada	Distoangulada	Invertida	Ectópica	Total		
Braquifacial	n	12	19	0	5	1	37	0,223
	%	32,40%	51,40%	0,00%	13,50%	2,70%	100,00%	
Mesofacial	n	5	27	1	4	3	40	
	%	12,50%	67,50%	2,50%	10,00%	7,50%	100,00%	
Dolicofacial	n	3	27	1	4	2	37	
	%	8,10%	73,00%	2,70%	10,80%	5,40%	100,00%	
Biotipo Facial		Posición de la tercera molar retenida en el cuadrante IV						p
		Vertical	Mesioangulada	Horizontal	Invertida	Ectópica	Total	
Braquifacial	n	10	20	1	1	1	33	0,080
	%	30,30%	60,60%	3,00%	3,00%	3,00%	100,00%	
Mesofacial	n	5	25	6	0	3	39	
	%	12,80%	64,10%	15,40%	0,00%	7,70%	100,00%	
Dolicofacial	n	3	26	8	0	1	38	
	%	7,90%	68,40%	21,10%	0,00%	2,60%	100,00%	

Test de chi cuadrado

## DISCUSIÓN

El propósito de esta investigación fue determinar la relación que existe entre la retención de terceros molares con el biotipo facial mediante radiografías panorámicas y cefalométricas de pacientes de ambos sexos entre las edades entre 18 a 36 años de edad. Para comprobar el biotipo de cada paciente, se emplearon medidas del análisis de Ricketts y el índice de VERT, tal como lo utilizaron Cerda-Peralta *et al*. ^24^, quienes indicaron que es uno de los análisis más estables y reproducibles para la definición del biotipo facial. Al referirnos a la relación existente entre biotipo facial y la frecuencia de terceros molares retenidos, nos referimos a que la frecuencia de estas piezas retenidas podría ser más predominante en algún biotipo facial que en otro. Por ejemplo, siguiendo las características de cada uno de ellos, los pacientes dólicofaciales, al tener una diferencia más acentuada en sentido vertical que en horizontal presentando arcadas más angostas. Además, podrían tener falta de espacios para las piezas dentales y eso estaría relacionado con que presenten una mayor probabilidad de retención de los terceros molares; sin embargo, esto fue descartado en nuestro estudio. 

Los resultados del presente estudio muestran que no hubo asociación significativa entre la posición del tercer molar y el biotipo facial, lo que muestra una discrepancia mínima entre biotipos y hace que los clínicos no predispongan una asociación entre estas dos variables. Estos resultados son similares con algunos estudios como el de Lindauer ^26^, en el cual hacen mención que tampoco hubo relación significativamente importante entre la retención del tercer molar y el apiñamiento anterior. Asimismo, Cerda-Peralta *et al*. ^18^^,^^24^ señalaron que existen otros factores diferentes de la edad y el biotipo facial, relacionados con aspectos físicos importantes como el dimorfismo sexual, que serían significativos, con diferencias marcadas en hombres y mujeres que podrían ser relevantes para determinar la retención de un tercer molar y otros factores relacionados con el aspecto físico.

Otras consideraciones importantes respecto a la impactación de estos dientes que se deben de tener en cuenta incluyen lo señalado por Nery *et al*. ^7^, quienes llegaron a la conclusión de que la retención de los terceros molares dependía directamente del tamaño de la arcada y el espacio destinado a cada pieza dentaria, más que el biotipo facial. Días Ribeiro *et al*. ^8^ hicieron mención que se puede evaluar la posición de estas piezas según la relación del tercer molar al segundo molar, y también según Castañeda *et al*. ^15^ que encontraron en la mayoría de estas piezas retenidas en el maxilar estaban en posición distoangulada, mientras que en la mandíbula estas piezas estaban mesioanguladas. Más allá, en nuestro estudio los resultados señalaron que no existe asociación entre la posición de los terceros molares retenidos y el biotipo facial. Esto podría deberse a estos otros factores etiológicos que tienen más influencia que el biotipo facial.

Finalmente, en nuestro estudio no se encontró relación significativa entre la frecuencia de retención de los terceros molares con el biotipo facial, además se encontró relación significativa entre el biotipo facial con relación al sexo de los casos revisados, igualmente no se encontró relación significativa entre la frecuencia de retención de los terceros molares y la edad de los pacientes.

**Figura 1:**
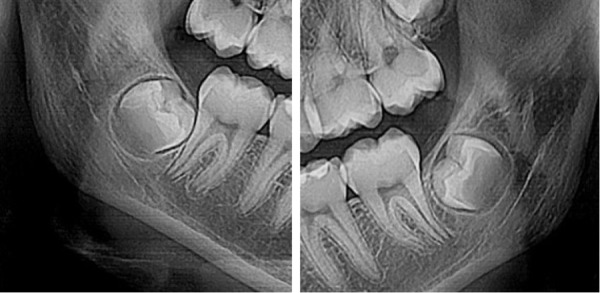
Coronas en evolución intraósea, tercera molar inferior derecha incluida 4.8 (A), tercera molar inferior izquierda incluida 3.8 (B).

**Figura 2:**
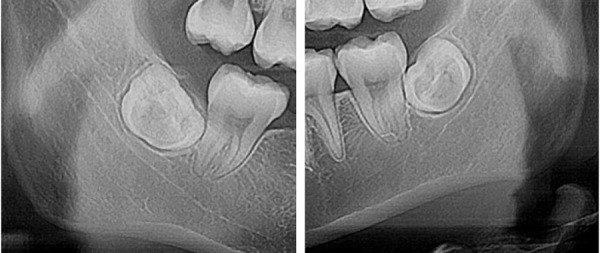
Coronas intraóseas, tercera molar inferior retenida en posición transversal 4.8 (A), tercera molar inferior izquierda retenida en posición transversal 3.8 (B).

**Figura 3:**
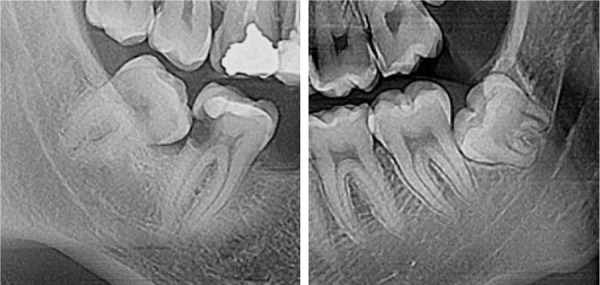
Tercera molar inferior derecha impactada 4.8 (A), tercera molar inferior izquierda impactada 3.8 (B).

**Figura 4:**
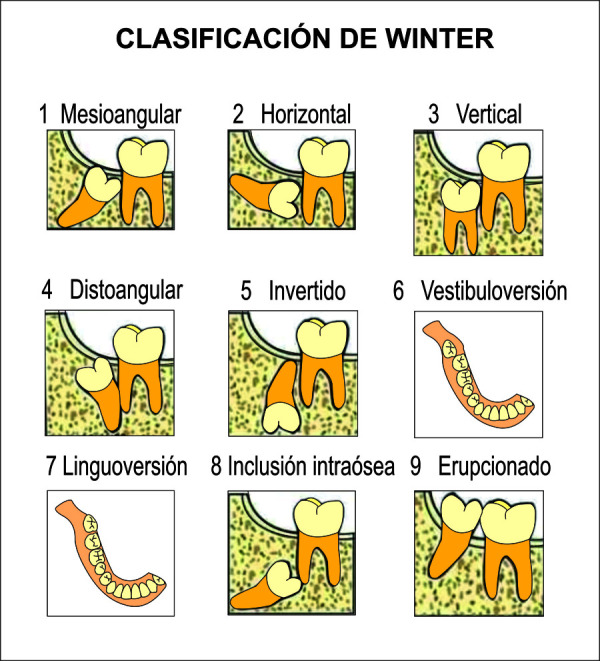
Clasificación según Winter

**Figura 5:**
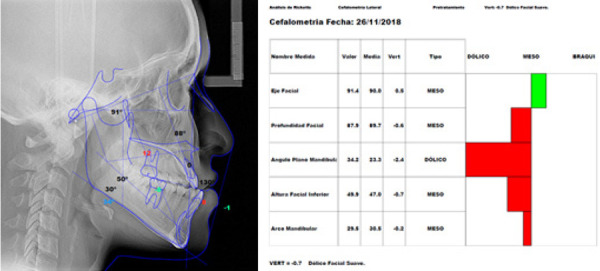
Radiografía cefalométrica con trazados del índice de VERT de Ricketts (A), valores de la cefalometría concluyendo en dolicofacial (B).

**Figura 6:**
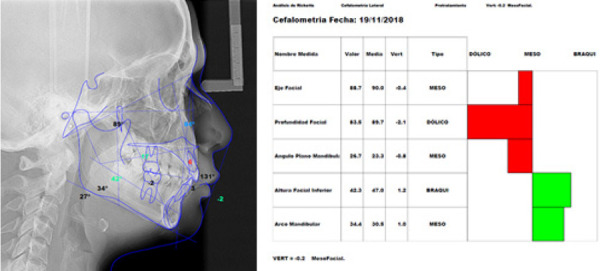
Radiografía cefalométrica con trazados del índice de VERT de Ricketts (A), valores de la cefalometría concluyendo en mesofacial (B).

**Figura 7:**
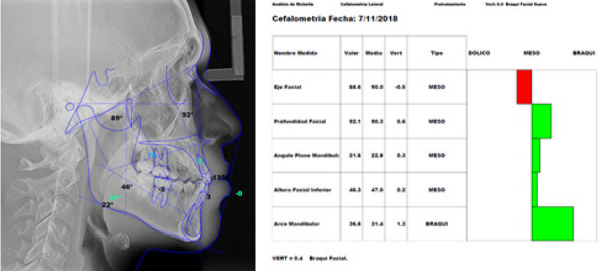
Radiografía cefalométrica con trazados del índice de VERT de Ricketts (A), valores de la cefalometría concluyendo en braquifacial (B).

## CONCLUSIONES

La posición de los terceros molares retenidos no está asociada con el biotipo facial. Este resultado debería ser tenido en cuenta por los clínicos al momento de realizar un diagnóstico de impactación.

## Fuente de financiamiento:

Autofinanciado
